# Effectiveness of different physical activity programs in improving older adults’ physical capacities: a randomized controlled trial

**DOI:** 10.3389/fphys.2025.1540776

**Published:** 2025-05-29

**Authors:** Carolina A. Cabo, Víctor Hernández-Beltrán, Orlando Fernandes, Cláudia Mendes, José M. Gamonales, Mário C. Espada, José A. Parraca

**Affiliations:** ^1^ Departamento de Desporto e Saúde, Escola de Saúde e Desenvolvimento Humano, Universidade de Évora, Évora, Portugal; ^2^ Comprehensive Health Research Centre (CHRC), University of Évora, Évora, Portugal; ^3^ Instituto Politécnico de Setúbal, Escola Superior de Educação, Setúbal, Portugal; ^4^ Sport Physical Activity and Health Research and Innovation Center (SPRINT), Rio Maior, Portugal; ^5^ Optimization of Training and Sports Performance Research Group, Faculty of Sport Science, University of Extremadura, Cáceres, Spain; ^6^ CBIOS-Universidade Lusófona’s Research Center for Biosciences and Health Technologies, Lisbon, Portugal; ^7^ Faculty of Health Sciences, University of Francisco de Vitoria, Madrid, Spain; ^8^ Programa de Doctorado en Educación y Tecnología, Universidad a Distancia de Madrid, Madrid, Spain; ^9^ Life Quality Research Centre (CIEQV-Leiria), Rio Maior, Portugal; ^10^ Center for the Study of Human Performance, Faculdade de Motricidade Humana, Universidade de Lisboa, Lisboa, Portugal

**Keywords:** aqua aerobic, older people, pilates, physical capacities, sensorimotor training

## Abstract

**Introduction:**

As people age, maintaining physical fitness becomes essential for preserving independence, preventing falls, and improving overall quality of life. Physical activity (PA) mitigates the physical decline associated with aging, enhancing balance, strength, flexibility, and coordination. Effective exercise programs for older people should address age-related physical challenges while remaining safe and accessible.

**Objective:**

This study aims to identify the most effective PA program to enhance the physical capacities of older people. By comparing training modalities such as Pilates, Aqua Aerobic, and Sensorimotor training, the study evaluates their impact on key physical abilities to determine the optimal program for promoting functional independence and reducing injury risk in older people.

**Methods:**

This study examined 153 participants, divided into a Control Group (N = 44), Sensorimotor Group (N = 46), Aqua Aerobic Group (N = 41), and Pilates Group (N = 22). Over a 24-week intervention, physical capacities were assessed using the Rikli and Jones battery for strength and flexibility and the timed-up-and-go test for agility and speed. Data were collected pre- and post-intervention. To analyze the changes, we used Student’s T-test and Cohen’s d for effect size (ES), with statistical significance set at *p* < 0.05. Additionally, ANOVA was applied to examine the main effects of time, group, and their interaction, with Partial Eta Squared used to determine the effect size (ES) in these comparisons.

**Results:**

The Sensorimotor Group showed significant gains in all tests (*p* < 0.05), while the Aqua Aerobic Group showed improvements in the Stand and Sit with and without Leaning (*p* < 0.001), Forearm Flexion (*p* = 0.005), and Reach Behind your Back (*p* = 0.002). In contrast, the Control and Pilates Groups did not exhibit significant improvements in any of the assessed variables. The analysis of the moment*group interaction effect revealed significant differences among the groups, except for the Timed Up and Go (TUG) test.

**Conclusion:**

In conclusion, sensorimotor and aquatic training significantly improved physical function, especially balance, strength, and mobility, in older people.

## 1 Introduction

By 2050, the number of people aged 80 and older is expected to reach 434 million—a threefold increase. Globally, the population over 60 is growing at a rate of 3% per year, outpacing the growth of younger age groups. Projections indicate that by 2050, 22% of the global population will be older people (World Health Organization, 2020). This demographic shift represents a significant social revolution of the 21st century, with profound social, political, and economic implications.

As people age, molecular and cellular deterioration affects all bodily systems, leading to a decline in physical and psychological health. However, physical activity (PA) has been shown to slow this decline by enhancing or maintaining intrinsic and functional capacities such as strength, balance, and flexibility. This makes PA a key factor in mitigating the effects of aging on bio-psychosocial functions, thereby improving the quality of life for the older people (Wickramarachchi et al., 2023). Additionally, the role of PA in enhancing health outcomes and reducing healthcare costs aligns with the objectives of current and future policy decisions (Duijvestijn et al., 2023).

A particular kind of training called sensorimotor training integrates the motor and sensory systems to enhance movement control and coordination. According to certain research, sensorimotor training may help healthy people with their postural sway, balance, and coordination. Research on its efficacy for senior citizens is, nevertheless, scarce. It is yet unknown if sensorimotor training results in a direct improvement in muscle strength, even though it can improve motor abilities. Therefore, more investigation is required to ascertain the precise effect of sensorimotor training on strength (Di Corrado et al., 2023; Varjan et al., 2024).

Joseph Pilates developed the Pilates method in the 1920s, and it is recognized as one of the most effective strategies for achieving the goals of Healthy Ageing due to its holistic approach. The method combines exercises that integrate the mind and body, requiring strength, flexibility, and trunk stability while also emphasizing breathing, posture, and muscle control (Kloubec, 2011). This versatility contributes to the method’s effectiveness, leading to psychomotor benefits and enhanced functional capacity, promoting greater independence and quality of life. In recent years, the body of research on Pilates has grown, with studies indicating its benefits for various populations, including older adults. Evidence suggests that Pilates is effective in improving balance, flexibility, muscle strength, and posture, which are essential for preventing falls and maintaining autonomy in daily activities (Pereira et al., 2022). Furthermore, recent systematic reviews have highlighted its positive impact on chronic conditions, such as low back pain, by improving core stability and reducing pain intensity (Patti et al., 2024). Despite these promising findings, further research is needed to explore its long-term effects on physical capacities in the older population, particularly in comparison with other exercise modalities.

Aquatic exercise has also gained popularity among older adults, as it minimizes or overcomes some of the limitations of land-based programs due to the unique properties of water, such as buoyancy and viscosity. These properties reduce joint stress while providing resistance, making water-based exercise a safe and effective alternative for individuals with mobility limitations or joint-related conditions. Additionally, evidence suggests that aquatic exercise programs are equally or even more effective than land-based ones in enhancing balance, strength, and overall wellbeing in older individuals (Farinha et al., 2021).

Given these considerations, key questions arise: Are sensorimotor training, Pilates, and aqua aerobics effective methods for promoting healthy aging? If so, how do they contribute to this goal? This study aimed to evaluate the effectiveness of sensorimotor training, Pilates, and aqua aerobics in enhancing physical capacities in individuals over 55 years of age. By comparing these exercise programs to a control group, we seek to determine which approach yields the most significant benefits for healthy aging. We hypothesize that each program will contribute to improved physical capacities, with distinct advantages depending on the training method.

## 2 Materials and methods

### 2.1 Design

The present paper is randomized controlled trial, examining the evolution of characters over a long time, and evaluating the physical capacity of older people after different PA programs (Montero and León, 2007).

### 2.2 Participants

**FIGURE 1:**
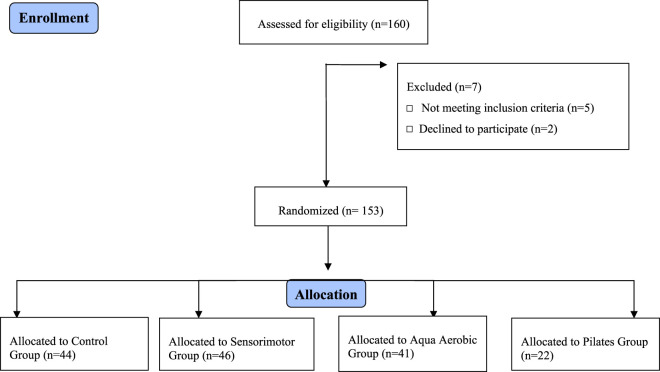
Study design and groups´ characterization.

Sample size calculations were performed using the G*Power 3.1.9.4 software (Kiel University, Kiel, Germany), selecting the statistical test to compare the differences between groups. Thus, accepting an alpha risk of 0.05 and assuming a moderate ES of 0.4, a total of 112 participants were sufficient to reach a minimum potency of 95%. From a total of 160 regular participants in the program, this study included 153 subjects aged between 55 and 80 years. The study population comprised individuals residing in Portugal, specifically in the municipality of Almada. All participants were community-dwelling older adults who met the study’s inclusion criteria. Participants were randomly assigned to the experimental or control group using computer-generated randomization with stratification based on age and baseline functional capacity. To minimize expectancy bias, participants were informed about the general objectives of the study but blinded to the specific purpose of the group comparisons. Additionally, researchers responsible for data analysis were blinded to group allocation. A parallel-group randomized controlled trial was conducted, with a 6-month intervention phase. Figure 1 describes the study design and particularities associated with each group.

The participants were randomly assigned to the four groups shown in Table 1 (Control Group N = 44; Sensorimotor Group N = 46; Aqua Aerobic Group N = 41; and Pilates Group N = 22). No dropouts occurred, and subjects were evaluated previously and shortly after the end of the intervention program. Table 1 displays the characteristics of the sample to provide context and frame the sample used in the study.

**TABLE 1:** Characteristics of the sample selected to perform the study.

	Gender	N	Age (year)		Weight (kg)		Height (m)		BMI (kg/m^2^)	
			Mean	SD	Mean	SD	Mean	SD	Mean	SD
Control Group	Female	37	72.6	5.47	68.2	11.6	1.56	0.05	28.1	4.82
	Male	7	79.6	6.08	80.1	14.3	1.68	0.05	28.2	4.24
Sensorimotor Group	Female	36	71.8	7.02	66.8	14.2	1.55	0.06	27.7	5.41
	Male	10	74.7	6.07	74.3	13.5	1.68	0.07	26.1	3.18
Aqua Aerobic Group	Female	28	73.7	6.73	69.7	14.1	1.55	0.05	29.0	5.82
	Male	13	74.9	6.55	81.3	13.1	1.67	0.07	28.9	3.51
Pilates Group	Female	18	70.6	4.53	65.6	9.52	1.59	0.05	26.0	3.64
	Male	4	72.5	5.45	74.1	9.55	1.64	0.03	27.5	2.46

Note. Kg: Kilograms; m: Meters, BMI: body mass index; SD: standard deviation.

Participants should meet the following inclusion criteria: (1) aged between 55 and 80 years old; (2) without prothesis (except dental prosthesis); and (3) who had no surgical interventions 6 months before data collection. Exclusion criteria: (1) musculoskeletal diagnosis; (2) problems in locomotion; (3) psychiatric diseases and neurological disorders; and (4) clinical cardiovascular diagnosis. These inclusion and exclusion criteria were established to prevent any damage associated with the participants because the intervention focused on a sensorimotor training program under different situations.

### 2.3 Ethics

The Ethics Committee of the University of Évora approved this project (approval number: 21040). The study was registered with the Clinical Trials.gov PRS Protocol Registration and Results System (Registration Number: NCT05398354; https://www.clinicaltrials.gov/ct2/show/NCT05398354?term=NCT05398354&draw=2&rank=1.

Each participant provided informed consent before participating, according to the Helsinki Declaration for Human Studies.

### 2.4 Intervention programs

**FIGURE 2:**
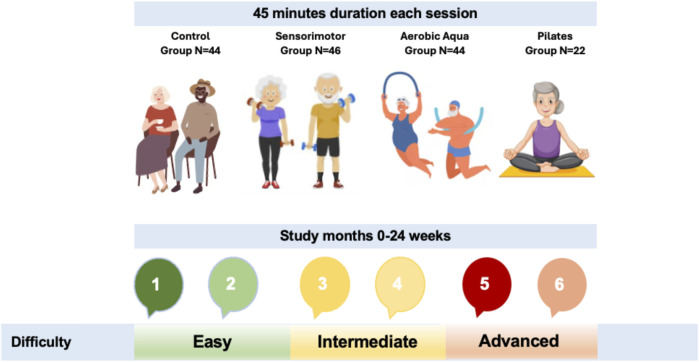
Study timeline graph.

The sensorimotor program (Figure 2) was conducted for 6 months, with a frequency of twice a week. As the program progressed, there was a progressive increase in the load. To this end, the session was divided into three levels of intensity: easy (no external load during the first 8 weeks), intermediate (application of external load: eic bands, shin guards and free weights, from the 9th to the 16th week) and advanced (increase in external load for the previous level, from the 17th to the 24th week). Each month, a different type of session was developed. Each session lasted 45 min, divided into three phases: the initial phase (10 min), consisting of a 5-min walk followed by a joint warm-up; and the fundamental phase (25 min), where the patients worked on a corresponding circuit of exercises. This circuit consisted of four cycles, with eight exercises each (50 s on, 15 s off); and a return to calm (10 min), where muscle stretching was performed.

The aqua aerobic program was conducted for 6 months, with a frequency twice a week. An aqua aerobics plan focused on improving mobility, strength, balance, and cardiovascular health while minimizing injury risks. The sessions began with a 10-min warm-up, involving gentle walking or marching in shallow water, along with arm circles and shoulder rolls to prepare the body. The main workout lasted 20 min, starting with 5 min of water walking or jogging, both forward and backward, to boost balance and coordination. This was followed by leg lifts, alternating between side and front lifts, aimed to increase strength and flexibility. Arm curls using water resistance were then performed to build upper body strength. Seated bicycle movements, while supporting the pool wall, target core and leg strength. The sessions terminated with a 10-min cool down of slow walking and gentle stretching to relax muscles and improve flexibility.

The Pilates program was conducted for 6 months, with a frequency of twice a week. Prioritizing safety, flexibility, strength, and balance. The sessions began with a gentle warm-up, incorporating deep breathing and light stretches to prepare the body. Participants then moved into core strengthening exercises, such as pelvic tilts and seated leg lifts, to enhance stability. Following this, they engaged in gentle spine articulation, like the cat-cow stretch, to promote spinal flexibility. Side-lying leg lifts and clamshells were included to strengthen the hips and glutes. Balance exercises, such as standing on one leg while holding onto a chair for support, were conducted to improve stability. The sessions ended with a cool down, featuring seated forward bends and deep breathing to relax the muscles and promote a sense of wellbeing.

The Control Group did not participate in any activity during the intervention period but underwent assessments both before and after the intervention for comparison with the other groups. This allowed for an evaluation of the effects of the Sensorimotor Group, aerobic aqua group, and Pilates Group by providing baseline and post-intervention data without any external influence from physical activity.

A variety of tools were used to evaluate the studied variables. All measures were performed at baseline, at the end of the intervention program. Previous to the first measurement, all subjects participated in a familiarization phase to adapt themselves to the different instruments and assessments included in this study.

Main measures:

To assess the physical fitness of the participants, a tracksuit bottom was used and subjects were asked to remove accessories and any objects in their pockets. The following evaluations were carried out:

(I) Agility and execution speed were assessed through the Timed Up and Go (TUG) test, which consists of getting up from a chair, walking in a straight line 3 m away, and walking back and sitting down again (Barry et al., 2014; Bretan et al., 2013).
(II) Muscular endurance was evaluated by rising from the chair or bending and straightening for 30 s, during which the strength of the lower limbs involving the vastus medialis obliquus (VMO) and the vastus lateralis (VL) was also calculated (Lemmink et al., 2003).
(III) Upper limb strength was determined by the number of times that a determined weight can be lifted by performing a flexion-extension of the arms for 30s (Ginn et al., 2006).
(IV) Lower limb flexibility was assessed using the “sit and reach” test, in which the participants, from a seated position with one leg extended, slowly bent over, sliding their hands down the extended leg until they touched (or passed) their toes (Mayorga-Vega et al., 2014).
(V) Upper limbs flexibility was assessed using the “behind the back reach”, which consisted of measuring with a ruler the distance between (or the overlap of) the middle fingers behind the back (Ginn et al., 2006).

### 2.5 Statistical analysis

To assess the normality of the sample, the Kolmogorov-Smirnov test was conducted, revealing a p-value greater than 0.05. Therefore, the normality of the sample was assumed (Field, 2013). Parametric models were used to test the study’s hypotheses (O’Donoghue, 2012). The sample data was analysed to describe and understand it better. Average and standard deviation were used to look at how the data differed based on the gender of the participants. Afterward, a Student’s t-test was conducted to compare the differences between groups before and after the intervention, based on the first and second data collection. The ES of the differences was calculated using Cohen’s d proposal: trivial (0–0.2), small (0-2-0.6), moderate (0.6–1.2), large (1.2–2), very large (2–4), and extremally large (>4) (Cohen, 2013; Hopkins et al., 2009).

We employed a two-way repeated measures ANOVA to analyse the data. To comprehensively understand the effects of the intervention across different groups and time points, we conducted a two-way repeated measures ANOVA. This statistical method allowed us to evaluate the main effects of time (pre-vs. post-intervention), group (Control, Sensorimotor, Aqua Aerobic, Pilates), and the interaction effect between time and group. By using the two-way repeated measures ANOVA, we could determine whether the changes over time differed significantly between the groups, offering a more nuanced understanding of the intervention’s impact. This approach provided a robust analysis of the data, accounting for both within-subject and between-group variations. To analyse the ES (η2), we used Partial Eta Squared.

The software Jamovi (v2.3.18) was used to conduct statistical analyses. Statistical significance was determined at *p* < 0.05.

## 3 Results

Table 2 shows the differences between the pre- and post-intervention values according to the developed programs. These results reported that the users included in the intervention programs developed significant differences in the analyzed variables. In this line, the participants who participated in the Aqua Aerobic Group showed differences between the pre-and post-intervention values in certain variables (Stand and Sit with Leaning, Stand and sit without Leaning, Forearm flexion, and Reach Behind your Back (left). In this population, the ES was negative, small, and moderate.

**TABLE 2:** Inferential analysis of pre-and post-intervention values according to the different intervention programs.

Variables (pre-and post-values)	Control Group		Sensorimotor Group		Aqua aerobic group		Pilates group		Intervention effect Moment*Group	
	p value	ES	p value	ES	p value	ES	p value	ES	p value	ES (*η* ^2^)
Timed Up and Go (s)	0.450	0.115	**< 0.001**	0.575	0.085	0.276	0.456	0.162	0.395	0.020
Stand and Sit with Leaning (rep)	0.349	−0.142	**< 0.001**	−0.537	**< 0.001**	−0.688	0.413	−0.178	**0.039**	0.055
Stand and Sit without Leaning (rep)	0.905	−0.018	**< 0.001**	−0.743	**< 0.001**	−0.637	0.472	−0.156	**< 0.001**	0.115
Forearm Flexion (rep)	0.319	0.151	**0.002**	−0.490	**0.005**	−0.467	0.342	0.207	**0.003**	0.090
Sitting and Reaching (rep)	0.155	0.218	**< 0.001**	−0.653	0.203	0.202	0.677	0.089	**0.003**	0.090
Reach Behind your Back (right) (m)	0.277	0.165	**< 0.001**	−1.074	0.126	−0.244	0.307	−0.223	**< 0.001**	0.108
Reach Behind your Back (left) (m)	0.197	−0.197	**< 0.001**	−1.323	**0.002**	−0.505	0.409	−0.179	**< 0.001**	0.227

Note. S: seconds; rep: Repetitions; m: Meter; ES: effect size; *p* < 0.05. Cohen’s d for ES: trivial (0–0.2), small (0-2-0.6), moderate (0.6–1.2), large (1.2–2), very large (2–4), and extremally large (>4); Partial Eta Squared for ES *(η* ^*2*^ *)*: Small ES (0.01–0.06); # Medium ES (0.06–0.14); * Large ES (>0.14). Bold values with statistical significance.

The analysis of the moment*group interaction effect revealed significant differences between the groups, except in the Timed Up and Go (TUG) test, where no significant differences were found. Regarding ES, the Reach Behind Your Back test showed the highest values, with a moderate ES for the right side and a large ES for the left side. These findings highlight the substantial impact of the intervention on upper body flexibility, particularly in shoulder mobility.

**FIGURE 3:**
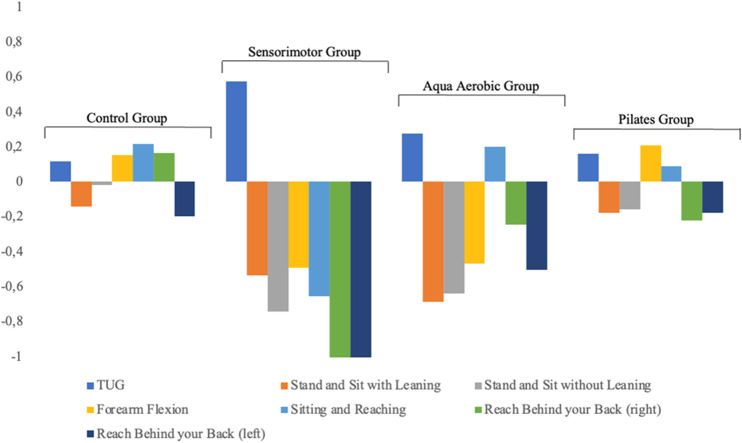
ES of the variables analyzed according to the different physical activity programs. Note. Timed Up and Go (TUG); Stand and Sit with Leaning (SSWL); Stand and Sit without Leaning (SSL); Forearm Flexion (FF); Sitting and Reaching (SR); Reach Behind your Back (right) (RBBR); Reach Behind your Back (left) (RBBL). ES is considered trivial (0–0.2), small (0.2–0.6), medium (0.6–1.2), large (1.2–2), very large (2–4), and extremely large (>4) (Cohen´s *d*).

Figure 3 shows the results related to the ES of the analysis (Hopkins et al., 2009), which improves the comprehension regarding the results obtained. The ES analysis revealed notable differences between the groups across various physical function tests. The sensorimotor training group showed the most substantial positive effects, particularly in flexibility and postural control measures. This was especially evident in the “Reach Behind Your Back” test (right: ES = −1.074; left: ES = −1.323) and the “Sitting and Reaching” test (ES = −0.653), indicating significant improvements in mobility and trunk flexibility. The aqua aerobic group also demonstrated moderate to strong effects, though generally lower than the sensorimotor group, especially in reach and upper-limb flexibility tasks. The Pilates group displayed only mild and localized effects, with slight improvements in upper-body strength (e.g., “Forearm Flexion,” ES = 0.207). As expected, the control group showed minimal or inconsistent effect sizes, with no meaningful improvements. These findings highlight the effectiveness of sensorimotor training in enhancing functional mobility and postural stability in older adults.

## 4 Discussion

This study aimed to examine the impact of various activity programs (such as Control Group, Sensorimotor Group, Aqua Aerobic, and Pilates) on the physical wellbeing of older adults. The study is characterized by a robust design, including a 6-month intervention period, which facilitated a meaningful assessment of physical fitness changes over time. The incorporation of sensorimotor, aerobic aqua, and Pilates groups allowed for a comprehensive evaluation of the effects of different exercise modalities. The main findings indicated that participants in the intervention programs demonstrated significant improvements across all analyzed variables. Additionally, those involved in aqua aerobics exhibited notable differences in specific variables (Stand and sit with leaning, Stand and sit without leaning, Forearm flexion, and Reach Behind your Back (left) before and after the intervention. The ES for this population ranged from small to moderate, with negative values. These results underscore the importance of targeting mobility, strength, and balance in the aging process. To improve the physical capacities of older adults, it is recommended to carry out a sensorimotor program that focuses on the main activities and movements required in daily routines. Such programs can improve balance, coordination, and effectiveness, ultimately enhancing quality of life. Furthermore, the well-defined physical fitness assessment methods ensured a homogeneous sample, thereby enhancing the external validity of the findings for the older adult population.

The results of the TUG test (Shumway-Cook and Woollacott, 2007) among different groups—sedentary individuals, those participating in Pilates, sensorimotor training, and aqua aerobics—can reveal significant insights into the impact of various PA programs on functional mobility. In the Control Group, significant values were not obtained. Participants in sensorimotor training typically show even greater improvements on the TUG test because this type of training targets balance and coordination, essential elements for mobility in older adults (Freire and Seixas, 2024). The integration of various sensory inputs during sensorimotor training is crucial for enhancing neuroplasticity, which allows the brain to reorganize and adapt to new stimuli. This process enables participants to react more effectively to external stimuli, potentially improving their balance and coordination. However, it is important to note that not all physical activities lead to a reduction in fall risk. According to Bianco et al. (2014) activities that specifically stimulate reaction time are most effective in reducing falls and injuries among older adults. These types of exercises promote faster and more accurate responses to environmental challenges, which are key factors in fall prevention. When analysing the data we obtained in our study, we found significant values with this activity (*p* < 0.001). Aqua aerobics and Pilates participants did not exhibit significant values.

The Control and Pilates groups performed more poorly on the Stand and Sit with Leaning test, often taking longer to complete the task compared to other groups. This delay indicates compromised strength, balance, and functional mobility, which are common among those who do not engage in regular PA (Maki and McIlroy, 1997). Lack of movement leads to muscle atrophy and reduced proprioception, significantly increasing the risk of falls and functional decline in this population (Cameron et al., 2016). In contrast, sensorimotor and aquatic aerobic groups showed even greater improvements.

The Forearm Flexion test (Rikli and Jones, 1999), when analyzing the results from the Control and Pilates groups, showed no significant findings. This population often experiences muscle atrophy and decreased overall physical performance, leading to challenges in daily tasks that require upper body strength (Cameron et al., 2016). Additionally, insufficient PA can contribute to a decreased quality of life and increased dependency on caregivers. In contrast, sensorimotor and aquatic aerobic groups showed significant results. Enhanced neuroplasticity and strength gained through sensorimotor training enable participants to perform the Forearm Flexion test more effectively, indicating improved upper body function and reduced fall risk. Aqua aerobics incorporates resistance exercises that targets the upper body, leading to improvements in muscle tone and strength.

Sensorimotor training contributes to improvements in the Sitting and Reaching test, although the primary focus of such training is balance and coordination. The dynamic movements and stability exercises involved in sensorimotor training indirectly enhance flexibility by improving muscle control and joint stability. Studies suggest that participants in sensorimotor training show increased overall mobility and flexibility, which can enhance their performance in flexibility tests like Sitting and Reaching (Pšeničnik Sluga and Kozinc, 2024). Compared to our study, only the Sensorimotor Group had significant results in this test.

Sensorimotor training positively impacted shoulder mobility, leading to moderate improvements in the “Reach Behind Your Back” test (Ahmad et al., 2019), although its primary focus was on balance and coordination. Participants in sensorimotor training programs often show a greater functional range of motion (ROM) than sedentary individuals, allowing them to perform better in shoulder flexibility tests (Freire and Seixas, 2024). In the Reach behind your backtest, both left and right, the Sensorimotor Group had significant results. In addition, on the Reach Behind your Back Left we also observed significant results from the Aqua Aerobic Group. Aqua aerobics offers significant benefits for shoulder flexibility due to the unique properties of water. The buoyancy of water reduces joint strain, allowing participants to perform a wider ROM with less discomfort. Aqua aerobics routines often incorporate shoulder and upper body movements, which improve joint mobility and flexibility over time (Song and Oh, 2022). As a result, older people who engage in aqua aerobics tend to perform well in the “Reach Behind Your Back” test, often surpassing sedentary individuals and showing comparable improvements to those in Pilates programs (Tsourlou et al., 2006).

The results suggest that the Sensorimotor and Aqua Aerobics groups demonstrated significant improvements in specific variables, highlighting the targeted benefits of these interventions. However, the negative effect sizes observed in certain measures, such as the Timed Up and Go (TUG) test, warrant further investigation. It remains uncertain whether these negative values stem from measurement errors, participant variability, or an actual decline in performance, requiring additional analysis to clarify these findings.

This study has several limitations. The small sample size in certain groups (e.g., Pilates, N = 22) may reduce statistical power, and the predominance of female participants limits generalizability to males. The sensorimotor training protocol lacks detailed descriptions of targeted muscle groups, repetition schemes, and risk management strategies. Additionally, the absence of long-term follow-up prevents assessment of sustained intervention effects. Potential confounders, such as diet, medication, and baseline fitness levels, were not controlled. The study’s age range (55–80 years) may also limit applicability to other populations. Lastly, the practical significance of small or negative effect sizes, particularly in tests like the Timed Up and Go (TUG), warrants further investigation.

Future studies should focus on increasing the sample size to enhance statistical power and improve the generalizability of the findings. A wider range of outcome measures, including cognitive function and quality of life assessments, should be incorporated to provide a more comprehensive understanding of the intervention effects. Long-term follow-up evaluations should be conducted to assess the sustainability of the benefits over time. It is also recommended to collect more detailed demographic data to facilitate the exploration of potential subgroup effects based on factors such as comorbidities and age ranges. Additionally, the clinical relevance of effect sizes should be further investigated alongside statistical significance to better interpret the practical implications of the findings.

As a future research direction, it is suggested to apply this program to a larger sample to allow for the extrapolation of results to the broader adult population. Furthermore, developing a program based on sensorimotor perception for individuals with specific comorbidities is proposed, aiming to determine whether the intervention leads to significant improvements in motor skills affected by these conditions.

## 5 Conclusion

This study highlights the effectiveness of exercise interventions for older adults, with sensorimotor and aquatic training demonstrating distinct benefits. The sensorimotor group showed significant improvements in balance, coordination, and strength, while the aquatic aerobics group enhanced lower body strength, mobility, and flexibility. In contrast, the Control and Pilates groups showed no significant gains, reinforcing the advantages of sensorimotor and aquatic training.

Despite the promising results, limitations such as small sample size, gender imbalance, and lack of long-term follow-up affect generalizability. Future studies should address these factors to confirm long-term benefits. Overall, sensorimotor training appears to be the most effective for enhancing overall physical function in older adults.

## Data Availability

The original contributions presented in the study are included in the article/supplementary material, further inquiries can be directed to the corresponding author.
